# Mycotoxins—Biomonitoring and Human Exposure

**DOI:** 10.3390/toxins13020113

**Published:** 2021-02-03

**Authors:** Kristina Habschied, Gabriella Kanižai Šarić, Vinko Krstanović, Krešimir Mastanjević

**Affiliations:** 1 Department of Process Engineering, Faculty of Food Technology Osijek, Josip Juraj Strossmayer University of Osijek, 31000 Osijek, Croatia; vkrstano@ptfos.hr (V.K.); kmastanj@gmail.com (K.M.); 2 Department of Agroecology and Environment Protection, Faculty of Agrobiotechnical Sciences Osijek, Josip Juraj Strossmayer University of Osijek, 31000 Osijek, Croatia; gabriella.kanizai@fazos.hr

**Keywords:** mycotoxins, biomonitoring, human health, exposure

## Abstract

Mycotoxins are secondary metabolites produced by fungal species that commonly have a toxic effect on human and animal health. Different foodstuff can be contaminated and are considered the major source of human exposure to mycotoxins, but occupational and environmental exposure can also significantly contribute to this problem. This review aims to provide a short overview of the occurrence of toxigenic fungi and regulated mycotoxins in foods and workplaces, following the current literature and data presented in scientific papers. Biomonitoring of mycotoxins in plasma, serum, urine, and blood samples has become a common method for determining the exposure to different mycotoxins. Novel techniques are more and more precise and accurate and are aiming toward the simultaneous determination of multiple mycotoxins in one analysis. Application of liquid chromatography (LC) methodologies, coupled with tandem mass spectrometry (MS/MS) or high-resolution mass spectrometry (HRMS) has become a common and most reliable method for determining the exposure to mycotoxins. Numerous references confirm the importance of mycotoxin biomonitoring to assess the exposure for humans and animals. The objectives of this paper were to review the general approaches to biomonitoring of different mycotoxins and the occurrence of toxigenic fungi and their mycotoxins, using recent literature sources.

## 1. Overview

Food safety has become an important term for authorities and consumers. The aim is to keep the consumers safe from any harmful compounds and to ensure the producers from economical losses in case of an outbreak of contaminants in the production chain. The recommendations and the measures taken by the companies and the legal bodies are based on risk evaluations reported by food safety authorities [1]. Current regulations are established on scientific opinions given by eminent institutions such as FAO/WHO Joint Expert Committee on Food Additives of the United Nations (JECFA) and the European Food Safety Authority (EFSA). This includes the involvement of AOAC International (Association of Official Analytical Chemists) and the European Standardization Committee (CEN) who are obligated to monitor and implement the requirements for adequate sampling and analytical methods [2]. The important stages of risk evaluation studies are to identify and characterize contaminants and to evaluate the exposure to certain hazardous materials [2,3,4]. This often means the implementation of long-term monitoring of the occurrence of concerning substances in food. Food contaminants consist of different compounds. But this review will be limited to the most common contaminants—mycotoxins (single or mixed) and other toxins produced by various fungal species [1], some of which belong to the genus *Aspergillus, Penicillium, Fusarium,* and *Alternaria* [5]. Mycotoxins and general exposure to their effect have become a major concern for the scientific and popular community. Mycotoxins are a big group of compounds, with a range of chemical structures and toxicological properties [6]. The most common mycotoxins included in legislation belong to several types: aflatoxins (AFs) and ochratoxins (OT’), fumonisins (FBs), trichothecenes and zearalenone (ZEA), patulin (PAT), and citrinin (CIT) [7]. The main food groups affected by fungal metabolites are different cereals, dried fruits, nuts, coffee, and spices [5]. Well-developed strategies including contamination control measures and improvements in processing technologies are efficient in mycotoxins prevention but despite these efforts, up to 80% of food still ends up contaminated by mycotoxins [8,9] and it has been estimated that cca. 25% of cereals worldwide are contaminated with mycotoxins [8]. Reduction of mycotoxins contamination via food processing (higher temperatures or high pressure) is minimal and allows them to linger in food items. Destruction in the gastrointestinal tract is also minimal. This is why they can act in such a harmful way and affect human and animal health. Their pronounced influence on the global economy is also tremendously important [10].

To regulate human exposure to food contaminants, especially mycotoxins, human biomonitoring (HBM) emerged as a recognized, efficient, and cost-effective method [11]. By applying HBM, it is possible to track exposure points and set minimum and maximum exposure limits. The research possibilities of HBM application can be used to understand the population range values and identify consumer groups and individuals or groups (e.g., geographically). This aims to detect higher exposures and also to confirm the regional and temporal variability for trends within a population [12]. To conduct valid research, several set-points regarding HBM need to be addressed. It is very important to provide a sufficiently sensitive and validated analytical method to obtain accurate measurements of a biomarker that correlates with the external dose [12]. The most commonly used biological material for HBM is urine, plasma, or blood. Urine is, however, preferred in field studies due to the noninvasive sampling method. This generally helps to gain higher acceptance by study participants [13]. The suitability of the biomarker or matrix greatly depends on the toxicokinetic profile of the studied compound. Detailed knowledge of the compounds’ toxicokinetics, such as general metabolism properties and excretion timeline, is necessary to translate the existence of HBM biomarker data into daily intake estimates [12].

Exposure to mycotoxins does not always have to be related to food consumption. There are studies [14,15,16,17,18,19,20,21,22] that explored the occurrence of mycotoxins in working or living environments and the results showed that exposure to mycotoxins can be related to these places too. This will be further discussed in Section 2.

## 2. Exposure

### 2.1. Food

The occurrence and co-occurrence of different mycotoxins are common in available foodstuffs. Table 1 and Table 2 contain basic information on the most relevant mycotoxins and fungi detected in different foodstuffs and environments. Exposure via food is practically inevitable since fungi can withstand different conditions that would normally be harmful to other microorganisms. The additional threat is that fungi produce mycotoxins when found in unfavorable conditions and mycotoxins also can “survive” the hostile environmental conditions during food processing, such as higher temperatures and high pressures. 

Dietary exposure to mycotoxins and chronic dietary exposure to a combination of mycotoxins is something that occurs daily [23]. As can be seen from Table 1, mycotoxins can be found in many types of food. Many reviews and original articles deal with worldwide exposure to mycotoxins [3,5,22,23,24,25,26]. 

The toxicological effects of different mycotoxins are reported in many in vivo studies. Descriptions of mycotoxicoses such as gastrointestinal problems, genotoxicity, estrogenicity, and even death can be found in numerous scientific reports [23,27,28,29]. 

From Table 1 it is visible that the most common mycotoxins are DON (deoxynivalenol), patulin, OTA and B (ochratoxin A and B), and AFs (aflatoxins). They can be found in everyday foods that are consumed all over the world. Mycotoxins can also be found in water, as confirmed by several studies. Special attention is paid toward the regulation of mycotoxins found in baby foods. Infants are susceptible to developing difficulties when in contact with these kinds of contaminants. To reduce the risk of developing health problems, many of the stated mycotoxins are regulated by the European legislative but many of them are still treated as emerging issues and their effect on human health is not yet well-described.

Besides the sites in which mycotoxins or fungi can reside, a novel approach to the study of mycotoxin exposure was described by Assunção et al. [60]. They published a review about the influence of climatic changes on aflatoxin exposure and overall human health issues related to these mycotoxins in Portugal. Global warming could result in higher numbers of hepatocellular carcinoma related to aflatoxins, as certain forecasts have predicted that aflatoxin contamination in European crops could become a food safety issue within the next 100 years. Battilani et al. [61] identified cereals and maize as crops susceptible to contamination due to climate change. Higher levels of mycotoxins detected in breakfast cereals have already confirmed the effect of climate change in this respect on cereal-based foods [60,61]. This shows that global warming affects fungal metabolism, and that when such fungi encounter unfavorable conditions (warmer temperatures, drought, stress), they produce more mycotoxins.

### 2.2. Environment

As mentioned before, biomonitoring means not only a follow up on mycotoxin intake via food, but active monitoring of possible exposure through inhalation, from the environment. To investigate something like this, it is immensely important to implement a suitable analysis method. The most common source of bodily fluids for mycotoxin detection is urine [62]. The presence of mycotoxins in a certain environment, such as working or living, and repeated exposure to contamination can pose a serious threat to human health. It is immensely important to provide the appropriate working safety equipment and conditions for exposed workers. In the following sections, the emphasis will be put on different sources of mycotoxin contamination, the most common mycotoxins, and exposure potential. 

Although these studies did not measure mycotoxins it is important to know the fungal microbiome surrounding us. It is proven that these fungi synthesize mycotoxins and therefore a possibility of mycotoxin contamination in the human body is not to be taken lightly.

### 2.3. Occupational Exposure to Mycotoxins

Exposure can be related to workplaces, especially in the field of agriculture (farms) and the food industry (mills, bakeries) [15,17,62,64,66,67]. Working environments with poor ventilation, inappropriate protective clothing, and equipment [68,69] expose workers to a higher risk of contamination. The accurate risk assessment of mycotoxins in agricultural-related workers is an important tool in ensuring the health of workers. Exposure to mycotoxins in working environments relies on the same methods of assessment, measuring their residual levels (or specific metabolites) as biomarkers in biological fluids including blood and urine [62,70]. In such cases, it is important to analyze and compare the urine from potentially exposed subjects and those who are nonoccupationally exposed (i.e., a control group) [62].

However, according to Föllmann et al. [62], the measured biomarkers in urine samples from the two groups, control (with dietary intake only) and the mill workers, showed dominantly dietary mycotoxin exposure, concluding that inhalational exposure of mill workers, is presumably very low. Nevertheless, the possibility of contamination still exists and attention should be paid to preserve the health of workers whenever possible.

## 3. Mycotoxins, Biomarkers, and Matrix Analysis

Mycotoxins that can be harmful to humans are included in the legislation of many countries. And, as can be seen from Table 1, these are the most detected mycotoxins in marketed foods. Some of them are not as common but are very toxic and thus included in the legislation. Aflatoxins, ochratoxin A, zearalenone, deoxynivalenol, fumonisins, and the methods of their detection and biomonitoring are well described by [21]. Some of it will be conveyed in this section.

Combining information from web source [5,71] mycotoxin biomarkers can be detected using three approaches:

- direct-or exposure-based biomarkers are specific; standardized analytical methods (optimized and validated), mainly for parent compounds, because not many metabolites are available as reference substances.
- Indirect—indirect (or biomarkers of effect) are generally non-specific and represent structural or functional alterations produced in the body under exposure to certain drugs or toxins;
- Non-targeted—the determination of unknown mycotoxin derivatives

Easily accessible biological matrices (urine, serum, plasma and breast milk) are key elements in HBM. Urine samples are easily obtainable by non-invasive methods, it can contain biomarkers of different mycotoxins [13]. However, urine biomarkers are susceptible to daily variations in mycotoxin intake, demanding at least 24 h sampling. Urine volume is individual for each person so this results in changes in the concentration of excreted compounds in the samples. This discrepancy can be reduced by applying a common—but still questionable—method for adjusting the mycotoxin levels in creatinine concentrations [72,73]. Namely, it is still unclear if the mycotoxin/creatinine ratio is applicable for comparison between individuals since creatinine is influenced by many factors (muscle mass, sex, age, season, diet, etc.) [74].

Breast milk is a great point to estimate the exposure in breastfed babies to different mycotoxins, but it is used to monitor only lactating women.

Other bodily fluids, such as serum and plasma, require somewhat invasive methods and educated medical staff to be collected, but can contain higher levels of studied chemicals [75] and are often used in long-term exposure studies [76].

Some mycotoxins can end up in the blood unchanged, and some can go through different modifications while in the human (or animal) metabolic system [77,78,79,80,81,82,83]. To calculate the exposure to mycotoxins, the metabolized form, very often the biomarker, needs to be included in the calculation [84,85]. Examples of well-established biomarkers for follow up on mycotoxins exposure is the production of glucuronic acid conjugates from deoxynivalenol. As mentioned before, some mycotoxins are resilient to endogenous metabolism and may be detected in their original form. A good example of this is the non-polar fumonisins (FB), which show off low rates of absorption and metabolic activity [77,86,87,88]. Since dietary analyses are broadly utilized in assessing the intake of food contaminants in the general population, certain tools need to be employed to reduce and clarify the variance among populations. For that reason, recent reports point to an increased interest in using dietary biomarkers to detect possible analytical errors in external exposure estimates [89].

Due to the complexity of blood, plasma, and serum samples, matrix components might interfere in analyte retention. They can also affect to reducing purification, recovery, and method sensitivity when mass spectrometry (MS) detectors are used.

Table 3 shows the most common and efficient methods for the determination of different mycotoxins or their biomarkers. Depending on the mycotoxin, extraction methods vary (IAC—immunoaffinity columns, SPE—solid phase extraction, LLE—liquid-liquid extraction, QuECHERS^®^ —quick, easy, cheap, effective, rugged, and safe, etc.), but some methods include a practical dilute-shoot or Filter-shoot preparation which makes the analysis more simple. Sample volumes also differ (10–0.1 mL), depending on the method. Detailed information can be found in an extensive review by Escrivá et al. [90].

Aflatoxins are one of the most well-studied mycotoxins with a metabolism that is familiar to scholars. Four different aflatoxins aflatoxin B1 (AFB1), aflatoxin B2 (AFB2), aflatoxin G1 (AFG1), and aflatoxin G2 (AFG2) are often detected and quantified as foodstuff contaminants. However, AFB_1_ is the most important not only because it is abundant but because of its toxicity. Biochemical reactions (hydroxylation and epoxidation) occurring in metabolism determine the form it will end up in. Hydroxylation at C-7 leads to the formation of aflatoxin M1 (AFM1) and epoxidation at C-8, C-9 ends up either in endo- or in exo-configuration. This makes AFM1 the first described metabolite and validated biomarker of aflatoxin exposure with an average conversion rate of about 1.5% [147] and can be determined in different biological fluids such as urine and breast milk [148]. Epoxidized form of AFB1 reacts with DNA (exo-epoxide only), glutathione, or is hydrolyzed to AFB-diol. Further complex metabolism of AFB-DNA-adducts is described in [21], but in short, aflatoxin B1-N7-guanine (AFB1-N7-guanine) and aflatoxin B1-glutathione conjugates are excreted via urine as aflatoxin B1-mercapturic acid conjugates (Aflatoxin B1-N-acetyl-cysteine, AFB1-NAC) and can serve as biomarkers [149,150]. For blood samples, the aflatoxin biomarker is an aflatoxin-albumin adduct and is the most common matrix for aflatoxins detection in urine, breast milk, and blood. It is usually determined via high-performance liquid chromatography coupled with fluorescence detection (HPLC-FLD) [64,65,151,152,153]. A faster and cheaper method, enzyme-linked immunosorbent assays (ELISA) can also be applied in aflatoxin and aflatoxin-metabolite analysis in blood and urine. Xue et al. [154] reported an almost completely non-invasive and utterly simple AFB1-albumin adduct detection from dried blood spots.

Ochratoxin A is characterized as nephrotoxic and carcinogenic mycotoxin, often found in food and feed [30,155,156]. It can be found in human serum even after 30 days [157] and serves as a biomarker for ochratoxin A exposure [158]. Detection of ochratoxin A in urine is not as precise and accurate since only quantities of ingested OTA end up in urine (<3% per day) [157,159], but a less invasive method, many research data originate from values obtained from urine samples [81,140,160]. Continuous and current studies are investigating the metabolic pathways involving OTA [116,161,162] and its forms (hydroxylated ochratoxin A (4R-OH-OTA) and ochratoxin α (OTα)) [140,160,163]. OTA can be detected in blood plasma [163,164,165,166] and method improvement is ongoing [5,140]. OTA can also be detected in dried blood spots and quantified using HPLC-MS/MS technique [167,168]. ELISA can also be applied for the analysis of ochratoxin A in plasma and has been widely used for the screening of samples [169,170,171]. Orti et al. [172] reported the first method for the analysis of OTA in human urine, but with simultaneous detection of ochratoxin A and aflatoxin B1. Some scholars [13] worked toward the improvement of these methods. In 2010 Munoz et al. published the protocol for HPLC-MS/MS analysis of OTA and OTα in urine [140]. Several authors [173,174] reported working on a molecular imprinted polymers method which can be employed for OTA purification from urine. In 2020 a group of scholars [175] conducted research on mycotoxin exposure assessments in a multi-center European validation study by 24-h dietary recall and biological fluid sampling where they established a positive correlation between the ochratoxin A (and several other mycotoxins) levels, 24 h exposure time and serum. This could help scholars and professionals to identify chronic exposure biomarkers and to easier assess a single-time point exposure.

Zearalenone (ZEN) is a *Fusarium* toxin that exhibits estrogenic properties after ingestion. In the gastrointestinal tract, it undergoes both, phase I and phases II metabolism described in detail by Metzler et al. [176]. In short, it can be reduced to α- or β-zearalenol (α-/β-ZAN) with α-ZAN being predominantly found in humans and pigs while β-ZAN can be found in cows. Following biochemical reactions lead to forming α-/β-zearalanol (α-/β-ZAN). This form can be found in sheep [177]. Oxidative metabolism can result in the formation of 8-hydroxy ZEN which was confirmed in vivo in the liver and urine of rats dosed with ZEN but not yet in humans [178,179]. Glucuronic acid conjugates of ZEN, ZAN, α-/β-ZAN, and α-/β-ZAN are known but only ZEN-14-O-β-glucuronide (ZEN-14-GlcA) is detected in human urine [122,129,180]. Analysis of ZEN and its metabolites in blood and urine samples using the HPLC-MS technique is currently the most used method and many scholars are working on improvements [122,181,182,183].

Deoxynivalenol and its acetylated forms, 3-acetyl-deoxynivalenol (3-AcDON), and 15-acetyldeoxynivalenol (15-AcDON) are designated as the most prevalent mycotoxins [184]. However, plant metabolites relating to deoxynivalenol are also gaining popularity and significance among scientists. DON causes gastrointestinal problems and oxidative damage, inhibits DNA, RNA, and protein biosynthesis by interaction with ribosomes [185,186,187]. The metabolism of DON is studied in animals and a small amount of data exist considering human metabolism. DON needs a short time to be excreted from the body [188]. They reported that cca 30% of DON uptake is excreted via urine in 24 h, but 40% of DON in urine can be recovered as the unmodified mycotoxin [188]. DON metabolism results with its masked forms (deoxynivalenol- 3-O-β-glucuronide (DON-3-GlcA), deoxynivalenol-15-O-β-glucuronide (DON-15-GlcA) and deoxynivalenol-O-glucuronide (DON-7-GlcA), deepoxy DON (DOM-1), and its glucuronide, DON-3-sulfate are some successfully detected in humans [126,189,190,191,192,193,194]. Due to the polarity of DON, blood screening is of no importance for biomonitoring [188], but detection and quantification of DON in urine and its metabolites via the HPLC-MS/MS technique is a common and reliable method [129,182,188]. Reportedly, DON levels, after oral administration, in human samples of urine span from 0.003 to 0.008 µg/mL [109]. Urinary daily excretion of 35.2 µg DON was determined in humans after 49.2 µg of DON daily intake, representing 68.3% of the established DON provisional maximum tolerable daily intake (PMTDI) [131].

Type A trichothecenes—T-2 and HT-2 toxin, nivalenol (NIV), and fusarenon X (FUS-X) are also considered toxicologically relevant food contaminants. T-2 toxin is a type A trichothecene. It is a result of the metabolism of different *Fusarium* spp., and can be found in cereals and cereal-based products [195]. T-2 toxin is an inhibitor of protein synthesis and mitochondrial function and displays immunosuppressive and cytotoxic effects. Toxicity of HT-2 toxin (HT-2) has been less investigated because T-2 toxin is rapidly metabolized to HT-2 in vivo and for that reason, the Joint FAO/WHO Expert Committee on Food Additives (JECFA) reported that the toxic effects of T-2 and HT-2 cannot be differentiated. T-2 toxin is rapidly absorbed, as the other trichothecenes, and excreted in feces and urine [196]. On that note, HT-2, as the dominant compound in in vitro and in vivo studies, should be considered as the main T-2 biomarker in urine and plasma samples [197]. Hydroxylated products and glucuronide forms could also be used as biomarkers for T-2 exposure. Hence, several studies detected T-2 and HT-2 in human urine [130,133]. The lack of commercial reference standards only HT-2-4-glucuronide has been detected in human urine [130]. The implementation of novel methods should allow the detection of additional T-2 metabolites, especially the dominant metabolite, 3′-OH-HT-2. NIV is classified as a type B trichothecene. It is common in wheat [198] and displays immunotoxic, hematotoxic, myelotoxic, developmental, and reproductive toxicity properties. Exposure to dietary NIV has been associated with an increased incidence of oesophageal and gastric carcinomas in certain regions of China [199]. Since NIV is water-soluble it can be easily absorbed, distributed, and eliminated without accumulation in all investigated animals (rodents, pigs, and poultry) [200,201]. Scholars investigating NIV in human urine determined moderate-to-high NIV concentrations in the urine samples due to the limit of detection (LOD) used in their study (>4.0 ng/mL) [182]. NIV can presumably be excreted in the glucuronidated form, similar to DON (>90% DON-3-glucuronide and DON-15-glucuronide) [126]. In short, NIV was scarcely studied regarding its metabolic and toxicokinetic properties but its high similarity with DON molecules can indicate that NIV-glucuronides are yet to be identified. However, deepoxy-NIV can be found in feces. FUS-X is also a type B trichothecene that can be found in cereals. It usually co-occurs with DON and NIV, but in lower levels [202]. As reported by [203], its toxicity is more potent than other type B-trichothecenes. It affects hematopoietic tissues, the spleen, and the thymus, and exerts intestinal inflammation, inhibits protein synthesis, induces apoptosis, and alters genetic material causing cell cycle delays, chromosomal aberrations, and sister chromatid exchanges [204]. According to several studies, FUS-X is absorbed from the gastrointestinal tract of different animals and oral bioavailability is dependent on the particular species in question [205,206,207]. FUS-X is converted to NIV (>90%) in the liver and kidney. FUS-X biomarker analysis should focus on quantification of NIV both in urine and in plasma [208].

Fumonisins (FUM) also originate from *Fusarium* fungi. Even though more than 30 compounds are designated as fumonisins, the most familiar are fumonisin B1 (FB1) and fumonisin B2 (FB2). Fumonisins are classified as carcinogens and can cause different anomalies in children [86,209]. FB1 in urine is the basis for HBM [87,210], based on data retrieved from research on humans that showed a 0.12–2% excretion via urine which is enough since high amounts of FB1 can be found in food [87,211,212]. Analytical methods for the detection of FB1 are usually based on urine and are done via the HPLC-MS system. The oral bioavailability is generally below 5% for FB1 [171].

Multi-mycotoxin detection unites several fungal species (*Fusarium* sp. *Aspergillus*, *Penicillium*) and monitors the co-exposure to several mycotoxins. Saving money and adding a new dimension to BMH, this method is getting more attention. In 2010 Ahn et al. [117] developed a method for urine samples in which they detected AF, OTA, and FUM via HPLC-MS/MS. Rubert et al. [118] determined T2/HT-2, DON, FB1, FB2, AFB1, AFB2, AFG1, AFG2, ZEN, and OTA. Emerging mycotoxins and mycotoxin metabolites should be included in multi-mycotoxin HBM [213,214] and legislative frames. However, they demand an unspecific sample treatment [129,215] which makes the analysis that much difficult and complex.

Based on data collected from the consumption of contaminated foodstuffs and the average occurrence of the toxin, the exposure to certain contaminants can be derived by calculation [31]. However, the classical analytical approaches provide valuable data. The estimation of exposure via biomarkers correlates the direct exposure assessment with the dietary mycotoxins ingestions [22]. HBM is by definition the analysis of mycotoxin biomarkers in body fluids and tissues and serves to estimate the internal exposure of humans to food contaminants [216,217]. Assessing exposure through mycotoxin levels in biological matrices has a certain advantage when compared to solely analyzing the occurrence of toxins in food and combining the obtained data with information on food consumption. Namely, HBM can neglect the contamination source (orally or by inhalation) since this information is not important for this method. HBM also requires a single determination per person and food sampling methods and consumption data collection are not necessary. For that reason, HBM should be conducted continuously and worldwide [218].

Biomarkers, however, have to be validated to assure that they accurately represent the level of intake of the considered food, that the sample type and time of sampling are appropriate for the intended use, and that the analytical method is valid according to current standards. Validation of biomarkers is an important factor for analytical validity measured according to the prescribed standards as well as a matter of biological (nutritional) validity. Different variables affect the content of biomarker precursors in foods and subsequently their metabolism and kinetics in individuals. Therefore, validation criteria must refer to biological aspects of the biomarkers as well [219,220,221].

## 4. Exposure Assessment

Exposure assessment is a difficult and synergistic approach including all available data is crucial for a sound conclusion. According to several authors, HBM, in correlation with different dietary surveys can be more useful for confirmation of exposure to mycotoxins, because it connects exposure to certain foods but it can expose the influence of other factors (differences in exposure due to socioeconomic or regional factors) to the results [116,222,223]. In short, HBM can be more of use in human health and dietary studies, than its use in exact exposure assessment of daily intake [224]. Exposure assessment of daily intake from blood or urine concentration remains difficult unless the human toxicokinetics and inter-individual differences are better understood [116,224].

### 4.1. Relevant Strategies for Data Collection

To assess the shifts in mycotoxins exposure, food consumption surveys are regularly updated. However, increased consumption of nutty cereals or beer may increase the exposure to mycotoxins present in nuts and cereals (malt), such as deoxynivalenol, aflatoxins, or ochratoxin A. Monitoring studies and data collections are important for analyzing trends in mycotoxin occurrence in raw materials and foods. The timely and continuous follow-up results with an updated exposure assessment, which is very important for HBM studies, and most importantly can lead to appropriate reactions and reduction recommendations. There are several methods, described below, that ensure data collection for further processing.

Food consumption surveys are conceived as questionnaires filled by volunteers who individually and in detail reminiscence at least two days of their diet. Such data sets are useful in aiming the assessment of dietary exposure to certain mycotoxin in the general population [225,226]. The major EU institution for such assessments is the European Food Safety Authority (EFSA), which formed the Comprehensive European Food Consumption Database, also called EFSA Comprehensive Database [227]. Global assessments are done by the Food and Agriculture Organisation (FAO) of the United Nations. EFSA also implemented the food classification system named FoodEx1 which serves to codify all foods and beverages present in the database. FoodEx2 is an upgraded version and enables more precise reporting of consumption patterns. These databases are holding information about food consumption pattens of infants, toddlers, children, adolescents, adults, and the elderly for the different Member States. Complex statistical methods are applied and the amount of data resulting from these surveys is vast. However, these summary statistics is a useful and quick screening tool in assessing chronic and acute exposure to hazardous substances. EFSA uses the detailed underlying consumption data at the individual level to perform more refined exposure assessments, both acute and chronic. System for Food Contamination Monitoring and Assessment Program, commonly known as GEMS (Global Environment Monitoring)/Food operated by WHO implements the program in cooperation with a network of Collaborating Centers and acknowledged worldwide national institutions. WHO and FAO have actively worked toward obtaining as much new data and have recently developed a new database for Individual Food Consumption Data which provides summary statistics at three levels of food categorization and can be used for an indication of the dietary exposure at a national level.

#### Data Collection

Data collection for exposure assessment can be related to several methods described in the following sections.

Food monitoring studies aim to investigate the prevalence and concentration of various contaminants, in an ingredient or food [1]. The procedure of sampling includes a random collection of samples from various points in the supply chain. An important point, is that this allows for the tracking of food products and relates this tracking back to the producer. Sampling can be done over a designated time. Samples can also be provided by surveys which are frequently published in the literature and available to the public [228,229,230,231]. For such studies, classification and description of foodstuffs play an important role in exposure estimations for the general population based on the geographical origin. This also contributes to the diversification of consumers to sensitive groups in the population (infants or people with specific diets) [232]. Important parameters such as sample size, sampling strategy, and sample preparation have to be noted as they could influence the results [233]. Immunoassays can be utilized as a screening method and can be a useful tool in assessing exposure. If not to determine the presence of a compound then to exclude its presence above a certain limit.

Total diet studies evaluate food samples which collectively make up a sample of the whole diet. Samples are collected, prepared, and pooled into composite samples per food category, as described in Ref. [234]. In such studies it is important to include seasonality because some foods may contain various mycotoxins levels due to climatic conditions). Geographical variation [235] is also important to incorporate since it covers the potential geographical differences. According to food safety authorities, EFSA, WHO, and FAO, the food list should cover about 90% of the food intake, should be as close as possible to the actual whole diet, and should include beverages and drinking water [234]. Representative food items and food processing habits should be as close as possible to the habits of the investigated population. In a total diet-like study, the food items for which contamination levels of the relevant (group of) substances are expected are sampled separately [235].

EFSA employs two types of total diet studies [235]:

(a) total diet study for screening (limited number of composite food samples for common food categories). In the case of high exposures, further examinations are performed to identify the source.
(b) total diet study for refined exposure assessment (a large number of samples for smaller, more refined, food categories).

Duplicate diet studies aim to provide a copy of all food items and beverages as consumed by one person at a certain time, e.g., during a 24 h period. Such studies measure the actual exposure of consumers to compounds of interest, but the effects of food processing and preparation are also considered [236]. Duplicate diet studies have various different version. For example, where only a portion of the diet is collected, or where foods are collected based on standardized or average diets [1]:

(a) cyclic sub-portion duplicate diet,
(b) subpopulation duplicate diet,
(c) targeted food duplicate diet and
(d) the total population diet.

Some of the methods for exposure assessment are described in the Dietary Assessment: A resource guide to method selection and application in low resource settings, a detailed handbook issued by the FAO in 2018 [237].

### 4.2. Exposure Assessment

Exposure assessment is defined as the qualitative and/or quantitative evaluation of the likely intake of chemical agents via food as well as exposure from other sources if relevant [1]. According to several authors, to estimate the dietary exposure of humans to mycotoxins, it is important to manage as much information on prevalence and levels in foods as possible and to combine them with consumption data [3,237]. Strategies employed to assess exposure can detect acute or chronic exposure.

Point estimate—a single mycotoxin concentration is combined with a single input parameter for consumption. The result is a single exposure estimation with a high degree of uncertainty. Data on concentration commonly originates from a food monitoring study or a total diet study which makes the conduct of such studies considerably facile [1].

Observed individual mean—is defined as the mean mycotoxin concentration per food product, combined with the food consumption per day per consumer, averaged over the days available in the survey and, divided by the individual’s body weight [238] (average exposure/kg of bw/person/day).

A probabilistic approach is designated to assess acute and chronic exposure.

(a) acute exposure—can be assessed by combining daily individual consumption patterns from a food consumption survey with randomly selected levels per food product from a databank with mycotoxin levels in individual samples [1].
(b) chronic exposure—statistical models that use the same input as the observed individual mean approach (see the previous section) help in gaining exposure to the certain mycotoxin.

The positives and negatives for both methods are in detail described in [1].

Dietary exposure using duplicate diet studies conducts the analyses of the compounds, resulting in an actual exposure level per day for that individual. The collected food consumption data can be used to evaluate the possible sources of exposure. Acute exposure can be evaluated if duplicate portions are collected on one day per individual.

The heterogeneous distribution in the matrix, differences between geographical regions, climate, and processing methods can make the assessment of mycotoxins to be difficult and complicated. Low concentrations, co-occurrence, and biotransformation to modified forms also make this kind of research complicated.

## 5. Prospects and Conclusions

Human biomonitoring studies are relevant tools for monitoring the global health situation. As mentioned in the review, climatic changes will only enhance the relevance of biomonitoring since mycotoxins levels in cereals important for human consumption are expected to rise. Improvements of analytical methods and techniques will surely strive toward higher levels of precision and accuracy and novel, emerging mycotoxins will be defined and detected in lower and lower doses. Intensive research of metabolic pathways will lead to the discovery of new biomarkers whose detection and quantification could simplify and evade the invasive methods such as drawing blood. Finally, a combination of different strategies and methods, combining research fields and overlapping scopes, should result in much more efficient and wider data collection for future research and, most importantly, application in human health protection.

## Figures and Tables

**Table 1:** The general overview of the most common mycotoxins that can be found in different foods.

Food	Mycotoxin(s)	Source
Pistachio	Aflatoxins B1, B2, G1, G2	[30,31]
Peanuts	Aflatoxins B1, B2, G1, G2 Ochratoxin A	[30,31]
Almonds	Aflatoxins B1, B2, G1, G2	[30,31]
Dried figs		[31]
Cereals	Aflatoxins B1, B2, G1, G2 Ochratoxin A Deoxynivalenol Zearalenone Enniantins	[30,32,33,34]
Barley	Deoxynivalenol Beauvericin	[30,33]
Malt	Aflatoxins B1, B2, G1, G2 Ochratoxin A Patulin Deoxynivalenol	[24,30,35]
Wheat flour	Aflatoxins B1, B2, G1, G2 Ochratoxin A Deoxynivalenol	[30]
Cereal porridge	Aflatoxins B1, B2, G1, G2 Deoxynivalenol	[30]
Breakfast cereals	Aflatoxins B1, B2, G1, G2	[36]
Cornflakes and corn-based foods	Fumonisins Beauvericin	[37,38]
Rice	Total aflatoxins, Aflatoxin B1, Ochratoxin A Beauvericin	[39,40,41]
Baby food Baby fruit foods	Aflatoxins B1, B2, G1, G2 Patulin Beauvericin	[30,42]
Breast milk	Aflatoxin M1 Beavericin Dihydrocitrinone Alternariol monomethyl ether Enniatin A Enniatin B Ochratoxin A Ochratoxin alpha Ochratoxin B Sterigmatocystin	[25,43,44]
Dried milk	Aflatoxins B1, B2, G1, G2	[30]
Milk	Aflatoxin M1	[30,43,45]
Milk porridge	Aflatoxin M1	[30]
Cheese	Aflatoxin M1	[46]
Yoghurt	Aflatoxin M1	[31]
Fruit drink	Patulin	[30]
Fruit foodstuffs	Patulin	[30]
Pork meat	Ochratoxin A	[47]
Pork hams	Ochratoxin A	[47]
Wine	Ochratoxin A	[48]
Beer	Ochratoxin A, Deoxynivalenol, Sterigmatocystin	[24,49,50,51]
Coffee	Ochratoxin A	[52]
Cacao beans	Ochratoxin A	[53]
Chocolate	Ochratoxin A Aflatoxins	[26,54,55]
Spices	Ochratoxin A, Sum of Aflatoxin B1, Aflatoxin B2, Aflatoxin G1 and Aflatoxin G2	[31]
Portable water Surface water Groundwater Industrial effluents Wastewater	Zearalenone Aflatoxin B1, B2, G1 Ochratoxin A	[56,57,58,59]

**Table 2:** The most habitual fungi in different environments.

Fungi	Environment	Fungal Domicile	Source
*Aspergillus* spp., *Eurotium* spp., *Cladosporium* spp., *Penicillium* spp.	Apartments	Air	[63]
*Penicillium* spp., *Aspergillus* spp., *Cladosporium* spp.	Basements	Air	
*C. herbarum*, *D. macrocarpa*, *P. crustosum*, *A. puulaauensis*, *P. italicum*, *P. waksmani* *R. stolonifera*	Library or archive	Air Surfaces	[64]
*Aspergillus* spp., *Cladosporium* spp., *Penicillium* spp., *Rhizopus* spp. *Trichoderma* spp.	Passenger vehicles	Filter or air-conditioning system	[65]

**Table 3:** Most common methods for determination of different mycotoxins or their biomarkers in various human samples

Mycotoxins	Matrix	Detection Technique	Source
OTA	Serum	ELISA CE-LIF (CE/laser-induced FD) HPLC-FD HPLC-FD LC-ESI-MS/MS	[91,92,93,94,95,96,97,98,99]
AFB1	Serum	ELISA	[70]
OTA, OTα	Serum	HPLC-FD	[34]
OTA	Urine	HPLC-FD HPLC-ESI-MS/MS	[100,101,102]
OTA, OTα	Urine	HPLC-FD	[103]
AFB1-N7-Gua	Urine	HPLC-ESI-MS/MS	[104]
DON-GlcA	Urine	LC-MS/MS	[105]
STG	Urine	LC-MS/MS	[106]
DON, DOM-1	Urine	LC-MS/MS	[107]
DON, DON-GlcAs	Urine	LC-MS/MS	[108]
DON, DOM-1, DOM-1-G, DON-G1, DON-G2	Urine	HPLC-APCI-MS/MS	[109]
FB1, FB2	Urine	HPLC-ESI-MS/MS	[110]
ZON, α-ZOL, β-ZOL, ZAN, α-ZAL, β-ZAL	Urine	HPLC-EC	[111]
CIT, HO-CIT	Urine	LC-MS/MS	[112,113]
DON, DOM-1	Urine	LC-MS/MS	[114,115]
OTA, OTA-GlcA, OTA-sulfates	Urine	LC-MS/MS	[116]
AFM1, FB1, FB2, OTA, OTα	Urine	HPLC-ESI-MS/MS	[117]
AFB1, AFB2, AFG1, AFG2, OTA, DON, ZON, FB1, FB2, T-2, HT-2	Urine	LC-QTRAP-MS/MS	[118]
AFM1, OTA, DON, DOM-1, α-ZOL, β- ZOL, FB1	Urine	HPLC-Qtrap-MS/MS	[119]
DON, NEO, AFB1, AFM1, HT-2, HT2, OTA, OTα, ZON, α-ZOL, β-ZOL, FB1	Urine	LC-MS/MS	[120]
DON, OTA, FB1, AFB1, ZON, T-2, HT-2, AFB1, CIT, DOM, DON-2-GlcA, ZON-14-GlcA, α-ZOL, β-ZOL, 4-OH-OTA, OTα, AFM1, AFB1-N7-Gua	Urine	LC-MS/MS	[121]
DON, DON-3-GlcA, DON-15-GlcA, DOM-1, NIV, T-2, HT-2, ZON, ZON-14-O-GlcA, α-ZOL, β-ZOL, FB1, FB2, OTA, AFM1	Urine	HPLC-ESI-MS/MS	[122]
DON, DON-3-GlcA, DON-15-GlcA, ZEN, ZEN-14-GlcA.	Urine	LC-MS/MS	[123]
DON, DOM-1, AFM1, FB1, ZON, α-ZOL, β-ZOL, OTA	Urine	UPLC-MS/MS LC-QTrap MS/MS UPLC-API 5000 MS/MS	[124]
AFM1, OTA, FB1, DON, DON-GlcAs, FB2, DOM-1, ZON, ZON-14-GlcA, α-ZOL, β-ZOL, T-2, HT-2, NIV	Urine	LC-MS/MS	[125]
AFB1, AFB2, AFG1, AFG2, AFB1-N7-gua, AFM1, CIT, DON, DON-3-GlcA, DOM-1, FB1, HFB1, OTA, OTα, 4-OH-OTA, T-2, HT-2, ZON, ZON-14-GlcA, α-ZOL, β-ZOL	Urine	LC-MS/MS	[126,127]
AFB1, AFB2, AFG1, AFG2, AFM1, CIT, OH-CIT, DON, DON-3-GlcA, DON-15-GlcA, DOM-1, DOM-1-3-GlcA, 3-ADON, 3-ADON-15-GlcA, 15-ADON, 15-ADON-3-GlcA, DAS, FB1, FB2, FB3, FUS-X, OTA, OTα, T-2, HT-2, ZON, ZON-14-GlcA, α-ZOL, α-ZOL-7-GlcA, α-ZOL-14-GlcA, β-ZOL, β-ZOL-14-GlcA.			
AFB1, DAS, FusX, 3-AcDON, 15-AcDON, α -ZEL, β-ZEL, OTα, DOM-1, FB1, FB2, FB3, DON, ZEN, T2, HT2, DON-3-GlcA, DOM-GlcA, ZEN-14-GlcA, β-ZEL-7-GlcA, β-ZEL-14- GlcA, α-ZEL-7-GlcA, α-ZEL-14-GlcA, 15-AcDON-3-GlcA, 3-AcDON-15-GlcA, OTA, CIT and AFM1	Urine	LC-MS/MS	[128]
DON, DON-3-GlcA, T-2, HT-2, HT-2-4-GlcA, FB1, AFB1, AFB2, AFG1, AFG2, AFM1, ZON, ZAN, α-ZOL, β-ZOL, ZON-14-GlcA, ZAN-14-GlcA, α-ZOL-14-GlcA, β-ZOL-14-GlcA, OTA, OTα, ENN B, DH-CIT	Urine	LC-MS/MS	[129]
DON, DON-3-GlcA, T-2, HT-2, HT-2-4-GlcA, FB1, AFB1, AFB2, AFG1, AFG2, AFM1, ZON, ZAN, α-ZOL, β-ZOL, ZON-14-GlcA, ZAN-14-GlcA, α-ZOL-14-GlcA, β-ZOL-14-GlcA, OTA, OTα, EN B, DH-CIT	Urine	LC-MS/MS	[130]
DOM-1, DON, 3-ADON, FUS-X, DAS, NIV, NEO, HT-2, T-2, ZON, α-ZOL, β-ZOL, ZAN, α-ZAL, β-ZAL	Urine	GC-MS/MS	[131,132,133]
DON, DOM-1, 3-ADON, 15-ADON, ZON, α-ZOL, β-ZOL, ZAN, α-ZAL, β-ZAL	Urine	GC-MS/MS	[134]
OTA	Breast milk	HPLC-FLD	[135]
AFM1, OTA	Breast milk	HPLC-FD, ELISA	[136]
ZON	Breast milk	ELISA, HPLC-FD	[137]
DON, 3-ADON, NIV, FUSX, NEO, DAS, HT-2, T-2, ZON, α-ZOL, β-ZOL, FB1, FB2, FB3, EN A, EN A1, EN B, EN B1, BEA, AFB1, AFB2, AFG1, AFG2, AFM1, STG, OTA, OTα	Breast milk	UHPLC-HRMS	[138]
AFB1, AFB2, AFG1, AFG2, AFM1, OTA	Breast milk	HPLC-FLD LC-MS/MS	[139]
OTA, OT_	Serum urine	HPLC-FLD HPLC-ESI-MS/MS	[140,141]
CIT	Serum urine	HPLC-FLD	[141,142]
ENs, BEA	Serum urine	LC-MS/MS	[143]
OTA, OTB	Feaces	HPLC-FLD	[144]
TCT, AFs, OTA	Urine and nasal secreations	ELISA, Fluorometry	[145]
AFB1, AFB2, AFG1, AF2, AFM1, AFM2, OTA, DON, NIV, T-2, HT-2, 3-ADON, 15-ADON, NEO, FUS-X, DAS, MAS, ZON, ZAN, α-ZOL, β-ZOL, α-ZAL, β-ZAL, T-2 triol, T-2 tertraol, DOM-1, FB1, FB2	Urine, blood, feces, saliva, nasal secretions, breast milk, amniotic fluid of pregnant women	HPLC-MS/MS	[146]

## References

[1] De Nijs M., Mengelers M.J.B., Boon P.E., Heyndrickx E., Hoogenboom L.A.P., Lopez P., Mol H.G.J. (2016). Strategies for estimating human exposure to mycotoxins via food. World Mycotoxin J..

[2] Van Egmond H.P., Schothorst R.C., Jonker M.A. (2007). Regulations relating to mycotoxins in food: Perspectives in a global and European context. Anal. Bioanal. Chem..

[3] Brera C., Debegnach F., De Santis B., Di Ianni S., Gregori E., Neuhold S., Valitutti F. (2014). Exposure assessment to mycotoxins in gluten-free diet for celiac patients. Food Chem. Toxicol..

[4] Marin S., Ramos A.J., Cano-Sancho G., Sanchis V. (2013). Mycotoxins: Occurrence, toxicology, and exposure assessment. Food Chem. Toxicol..

[5] Arce-López B., Lizarraga E., Vettorazzi A., González-Peñas E. (2020). Human biomonitoring of mycotoxins in blood, plasma and serum in recent years: A review. Toxins.

[6] Pulina G., Battacone G., Brambilla G., Cheli F., Danieli P.P., Masoero F., Pietri A., Ronchi B. (2014). An update on the safety of foods of animal origin and feeds. Ital. J. Anim. Sci..

[7] Alshannaq A., Yu J.-H. (2017). Occurrence, toxicity, and analysis of major mycotoxins in food. IJERPH.

[8] Eskola M., Kos G., Elliott C.T., Hajšlová J., Mayar S., Krska R. (2020). Worldwide contamination of food-crops with mycotoxins: Validity of the widely cited ‘FAO Estimate’ of 25%. Crit. Rev. Food Sci. Nutr..

[9] Schaarschmidt S., Fauhl-Hassek C. (2018). The fate of mycotoxins during the processing of wheat for human consumption. Compr. Rev. Food Sci. Food Saf..

[10] Bryden W.L. (2012). Mycotoxin contamination of the feed supply chain: Implications for animal productivity and feed security. Anim. Feed Sci. Technol..

[11] Choi J., Aarøe Mørck T., Polcher A., Knudsen L.E., Joas A. (2015). Review of the state of the art of human biomonitoring for chemical substances and its application to human exposure assessment for food safety. EFS3.

[12] Mally A., Solfrizzo M., Degen G.H. (2016). Biomonitoring of the mycotoxin zearalenone: Current state-of-the art and application to human exposure assessment. Arch. Toxicol..

[13] Solfrizzo M., Gambacorta L., Lattanzio V.M.T., Powers S., Visconti A. (2011). Simultaneous LC–MS/MS determination of Aflatoxin M1, Ochratoxin A, Deoxynivalenol, de-Epoxydeoxynivalenol, α and β-Zearalenols and Fumonisin B1 in urine as a multi-biomarker method to assess exposure to mycotoxins. Anal. Bioanal. Chem..

[14] Viegas S., Arezas P., Baptista J.S., Carneiro P., Cordeiro P., Costa N., Melo R., Miguel A.S., Perestrelo G. (2019). Biomarkers of Exposure—An Important Exposure Assessment Tool for Occupational Health Interventions.

[15] Viegas S., Assunção R., Martins C., Nunes C., Osteresch B., Twarużek M., Kosicki R., Grajewski J., Ribeiro E., Viegas C. (2019). Occupational exposure to mycotoxins in swine production: Environmental and biological monitoring approaches. Toxins.

[16] Viegas S., Assuncao R., Nunes C., Osteresch B., Twaruzek M., Kosicki R., Grajewski J., Martins C., Alvito P., Almeida A. (2018). Exposure assessment to mycotoxins in a portuguese fresh bread dough company by using a multi-biomarker approach. Toxins.

[17] Viegas C., Faria T., Caetano L.A., Carolino E., Quintal-Gomes A., Twaruzek M., Kosicki R., Viegas S. (2019). Characterization of occupational exposure to fungal burden in Portuguese bakeries. Microorganisms.

[18] Viegas C., Faria T., de Oliveira A.C., Caetano L.A., Carolino E., Quintal-Gomes A., Twaruzek M., Kosicki R., Soszczynska E., Viegas S. (2017). A new approach to assess occupational exposure to airborne fungal contamination and mycotoxins of forklift drivers in waste sorting facilities. Mycotoxin Res..

[19] Viegas S., Osteresch B., Almeida A., Cramer B., Humpf H.-U., Viegas C. (2018). Enniatin B and Ochratoxin A in the blood serum of workers from the waste management setting. Mycotoxin Res..

[20] Viegas S., de Oliveira A.C., Carolino E., Padua M. (2018). Occupational exposure to cytotoxic drugs: The importance of surface cleaning to prevent or minimise exposure. Arch. Ind. Hyg. Toxicol..

[21] Cramer B., Humpf H.-U., Viegas C., Viegas S., Gomes A., Täubel M., Sabino R. (2017). Human biomonitoring of mycotoxins for the detection of nutritional, environmental and occupational exposure. Exposure to Microbiological Agents in Indoor and Occupational Environments.

[22] Franco L.T., Ismail A., Amjad A., de Oliveira C.A.F. (2020). Occurrence of toxigenic fungi and mycotoxins in workplaces and human biomonitoring of mycotoxins in exposed workers: A systematic review. Toxin Rev..

[23] De Ruyck K., De Boevre M., Huybrechts I., De Saeger S. (2015). Dietary mycotoxins, co-exposure, and carcinogenesis in humans: Short review. Mutat. Res. Rev. Mutat. Res..

[24] Mastanjevic K., Sarkanj B., Krska R., Sulyok M., Warth B., Mastanjevic K., Santek B., Krstanovic V. (2018). From malt to wheat beer: A comprehensive multi-toxin screening, transfer assessment and its influence on basic fermentation parameters. Food Chem..

[25] Ezekiel C.N., Abia W.A., Braun D., Sarkanj B., Ayeni K.I., Oyedele O.A., Michael-Chikezie E.C., Ezekiel V.C., Mark B., Ahuchaogu C.P. (2020). Comprehensive mycotoxin exposure biomonitoring in breastfed and non-exclusively breastfed Nigerian children. medRxiv.

[26] Brera C., Debegnach F., De Santis B., Iafrate E., Pannunzi E., Berdini C., Prantera E., Gregori E., Miraglia M. (2011). Ochratoxin A in cocoa and chocolate products from the Italian market: Occurrence and exposure assessment. Food Control.

[27] Ahmed Adam M.A., Tabana Y.M., Musa K.B., Sandai D.A. (2017). Effects of different mycotoxins on humans, cell genome and their involvement in cancer (review). Oncol. Rep..

[28] Kamala A., Shirima C., Jani B., Bakari M., Sillo H., Rusibamayila N., De Saeger S., Kimanya M., Gong Y., Simba A. (2018). Outbreak of an acute aflatoxicosis in Tanzania during 2016. World Mycotoxin J..

[29] Probst C., Njapau H., Cotty P.J. (2007). Outbreak of an acute aflatoxicosis in Kenya in 2004: Identification of the causal agent. Appl. Environ. Microbiol..

[30] Malir F., Ostry V., Grosse Y., Roubal T., Skarkova J., Ruprich J. (2006). Monitoring the Mycotoxins in food and their biomarkers in the Czech Republic. Mol. Nutr. Food Res..

[31] Abrunhosa L., Morales H., Soares C., Calado T., Vila-Chã A.S., Pereira M., Venâncio A. (2016). A review of mycotoxins in food and feed products in Portugal and estimation of probable daily intakes. Crit. Rev. Food Sci. Nutr..

[32] Zinedine A., Soriano J.M., Moltò J.C., Mañes J. (2007). Review on the toxicity, occurrence, metabolism, detoxification, regulations and intake of zearalenone. An oestrogenic mycotoxin. Food Chem. Toxicol..

[33] Meca G., Zinedine A., Blesa J., Font G., Mañes J. (2010). Further data on the presence of *Fusarium* emerging mycotoxins enniatins, fusaproliferin and beauvericin in cereals available on the Spanish markets. Food Chem. Toxicol..

[34] Gruber-Dorninger C., Novak B., Nagl V., Berthiller F. (2016). Emerging mycotoxins: Beyond traditionally determined food contaminants. J. Agric. Food Chem..

[35] Mastanjevic K., Krstanovic V., Mastanjevic K., Sarkanj B. (2018). Malting and brewing industries encounter *Fusarium* Spp. related problems. Fermentation.

[36] Martins C., Assunção R., Cunha S.C., Fernandes J.O., Jager A., Petta T., Oliveira C.A., Alvito P. (2018). Assessment of multiple mycotoxins in breakfast cereals available in the portuguese market. Food Chem..

[37] Sydenham E.W., Shephard G.S., Thiel P.G., Marasas W.F.O., Stockenstrom S. (1991). Fumonisin contamination of commercial corn-based human foodstuff. J. Agric. Food Chem..

[38] Zinedine A., Meca G., Mañes J., Font G. (2011). Further data on the occurrence of *Fusarium* emerging mycotoxins enniatins (A, A1, B, B1), fusaproliferin and beauvericin in raw cereals commercialized in Morocco. Food Control.

[39] Buyukunal S.K., Kahraman T., Ciftcioglu G.R. (2010). Occurrence of AF, AFB1, OTA in rice commercialized in eastern Turkey. Pol. J. Environ. Stud..

[40] Toman J., Malir F., Ostry V., Grosse Y., Dvorak V., Roubal T., Neuchlova L. (2016). The occurrence of Ochratoxin A in white and parboiled rice. Czech J. Food Sci..

[41] Sifou A., Meca G., Serrano A.B., Mahnine N., El Abidi A., Mañes J., Zinedine A. (2011). First report on the presence of emerging *Fusarium* mycotoxins enniatins (A, A1, B, B1), beauvericin and fusaproliferin in rice on the Moroccan retail markets. Food Control.

[42] Mahnine N., Meca G., Elabidi A., Fekhaoui M., Saoiabi A., Font G., Mañes J., Zinedine A. (2011). Further data on the levels of emerging *Fusarium* mycotoxins enniatins (A, A1, B, B1), beauvericin and fusaproliferin in breakfast and infants cereals from Morocco. Food Chem..

[43] Muñoz K., Campos V., Blaszkewicz M., Vega M., Alvarez A., Neira J., Degen G.H. (2010). Exposure of neonates to Ochratoxin A: First biomonitoring results in human milk (Colostrum) from Chile. Mycotox Res..

[44] Munoz K., Blaszkewicz M., Campos V., Vega M., Degen G.H. (2014). Exposure of infants to Ochratoxin A with breast milk. Arch. Toxicol..

[45] Duarte S.C., Almeida A.M., Teixeira A.S., Pereira A.L., Falcao A.C., Pena A., Lino C.M. (2013). Aflatoxin M-1 in marketed milk in Portugal: Assessment of human and animal exposure. Food Control.

[46] Martins H.M., Magalhães S.A., Almeida I., Marques M., Guerra M.M., Bernardo F. (2007). Aflatoxin M1 determination in cheese by immunoaffinity column clean-up coupled to high-performance liquid chromatography. Rev. Port. Cienc. Vet..

[47] Rodrigues P., Silva D., Costa P., Abrunhosa L., Venâncio A., Teixeira A. (2019). Mycobiota and Mycotoxins in Portuguese pork, goat and sheep dry-cured hams. Mycotoxin Res..

[48] De Jesus C.L., Bartley A., Welch A.Z., Berry J.P. (2017). High incidence and levels of Ochratoxin A in wines sourced from the United States. Toxins.

[49] Varga E., Malachova A., Schwartz H., Krska R., Berthiller F. (2013). Survey of deoxynivalenol and its conjugates Deoxyniva-lenol-3-Glucoside and 3-Acetyl-Deoxynivalenol in 374 beer samples. Food Addit. Contam. Part A Chem. Anal. Control Expo. Risk Assess..

[50] Bertuzzi T., Rastelli S., Mulazzi A., Donadini G., Pietri A. (2018). Known and emerging mycotoxins in small- and large-scale brewed beer. Beverages.

[51] Peters J., van Dam R., van Doorn R., Katerere D., Berthiller F., Haasnoot W., Nielen M.W.F. (2017). Mycotoxin profiling of 1000 beer samples with a special focus on craft beer. PLoS ONE.

[52] Batista L.R., Chalfoun S.M., Silva C.F., Cirillo M., Varga E.A., Schwan R.F. (2009). Ochratoxin A in coffee beans (*Coffea Arabica*, L.) processed by dry and wet methods. Food Control.

[53] Raters M., Matissek R. (2005). Study on distribution of Mycotoxins in cocoa beans. Mycotox Res..

[54] Copetti M.V., Iamanaka B.T., Pereira J.L., Lemes D.P., Nakano F., Taniwaki M.H. (2012). Co-occurrence of Ochratoxin a and Af-latoxins in chocolate marketed in Brazil. Food Control.

[55] Kabak B. (2019). Aflatoxins and Ochratoxin A in chocolate products in Turkey. Food Addit. Contam. Part. B.

[56] Russell R., Paterson M. (2007). Zearalenone production and growth in drinking water inoculated with *Fusarium graminearum*. Mycol. Prog..

[57] Gromadzka K., Waœkiewicz A., Goliñski P., Œwietlik J. (2009). Occurrence of estrogenic mycotoxin Zearalenone in aqueous environmental samples with various NOM content. Water Res..

[58] Hartmann N., Erbs M., Wettstein F.E., Schwarzenbach R.P., Bucheli T.D. (2007). Quantification of estrogenic mycotoxins at the ng/L level in aqueous environmental samples using deuterated internal standards. J. Chromatogr. A.

[59] Mata A.T., Ferreira J.P., Oliveira B.R., Batoréu M.C., Barreto Crespo M.T., Pereira V.J., Bronze M.R. (2015). Bottled water: Analysis of mycotoxins by LC–MS/MS. Food Chem..

[60] Assuncao R., Martins C., Viegas S., Viegas C., Jakobsen L.S., Pires S., Alvito P. (2018). Climate change and the health impact of Aflatoxins Exposure in Portugal—An Overview. Food Addit. Contam. Part A Chem. Anal. Control Expo. Risk Assess..

[61] Battilani P., Toscano P., Van der Fels-Klerx H.J., Moretti A., Camardo Leggieri M., Brera C., Rortais A., Goumperis T., Robinson T. (2016). Aflatoxin B1 contamination in maize in Europe increases due to climate change. Sci. Rep..

[62] Föllmann W., Ali N., Blaszkewicz M., Degen G.H. (2016). Biomonitoring of mycotoxins in urine: Pilot study in mill workers. J. Toxicol Environ. Health Part A.

[63] Jakšić Despot D., Šegvić Klarić M. (2014). A Year-Round Investigation of Indoor Airborne Fungi in Croatia. Arch. Ind. Hyg. Toxicol..

[64] Gutarowska B., Skóra J., Stępień L., Twarużek M., Błajet-Kosicka A., Otlewska A., Grajewski J. (2014). Estimation of fungal contamination and mycotoxin production at workplaces in composting plants, tanneries, archives and libraries. World Mycotoxin J..

[65] Aquino S., de Lima J.E.A., do Nascimento A.P.B., Reis F.C. (2018). Analysis of fungal contamination in vehicle air filters and their impact as a bioaccumulator on indoor air quality. Air Qual. Atmos. Health.

[66] Mayer S., Engelhart S., Kolk A., Blome H. (2008). The significance of mycotoxins in the framework of assessing workplace related risks. Mycotoxin Res..

[67] Boonen J., Malysheva S.V., Taevernier L., Diana Di Mavungu J., De Saeger S., De Spiegeleer B. (2012). Human skin penetration of selected model mycotoxins. Toxicology.

[68] Saad-Hussein A., Taha M.M., Fadl N.N., Awad A.-H., Mahdy-Abdallah H., Moubarz G., Aziz H., El-Shamy K.A. (2016). Effects of airborne Aspergillus on serum Aflatoxin B1 and liver enzymes in workers handling wheat flour. Hum. Exp. Toxicol..

[69] Viegas C., Dias M., Almeida B., Caetano L.A., Carolino E., Gomes A.Q., Twaruzek M., Kosicki R., Grajewski J., Marchand G. (2020). Are workers from waste sorting industry really protected by wearing filtering respiratory protective devices? The gap between the myth and reality. Waste Manag..

[70] Degen G.H. (2011). Tools for investigating workplace-related risks from mycotoxin exposure. World Mycotoxin J..

[71] Mycotoxin Biomarkers: Ready for the Field?. https://www.allaboutfeed.net/Mycotoxins/Articles/2017/10/Mycotoxin-biomarkers-Ready-for-the-field-202618E/.

[72] Turner P.C., Flannery B., Isitt C., Ali M., Pestka J. (2012). The role of biomarkers in evaluating human health concerns from fungal contaminants in food. Nutr. Res. Rev..

[73] Ouhibi S., Vidal A., Martins C., Gali R., Hedhili A., De Saeger S., De Boevre M. (2020). LC-MS/MS methodology for simultaneous determination of patulin and citrinin in urine and plasma applied to a pilot study in colorectal cancer patients. Food Chem. Toxicol..

[74] Hernandez-Vargas H., Castelino J., Silver M.J., Dominguez-Salas P., Cros M.-P., Durand G., Le Calvez-Kelm F., Prentice A.M., Wild C.P., Moore S.E. (2015). Exposure to aflatoxin B 1 in utero is associated withDNA methylation in white blood cells of infants in The Gambia. Int. J. Epidemiol..

[75] Slobodchikova I., Vuckovic D. (2018). Liquid chromatography—High resolution mass spectrometry method for monitoring of 17 mycotoxins in human plasma for exposure studies. J. Chromatogr. A.

[76] Duarte S.C., Pena A., Lino C.M. (2011). Human ochratoxin A biomarkers—From exposure to effect. Crit. Rev. Toxicol..

[77] Hartinger D., Moll W. (2011). Fumonisin elimination and prospects for detoxification by enzymatic transformation. World Mycotoxin J..

[78] Assunção R., Vasco E., Nunes B., Loureiro S., Martins C., Alvito P. (2015). Single-compound and cumulative risk assessment of mycotoxins present in breakfast cereals consumed by children from Lisbon region, Portugal. Food Chem. Toxicol..

[79] Vidal A., Cano-Sancho G., Marin S., Ramos A.J., Sanchis V. (2016). Multidetection of urinary Ochratoxin A, Deoxynivalenol and its metabolites: Pilot time-course study and risk assessment in Catalonia, Spain. World Mycotoxin J..

[80] Ali N., Degen G.H. (2020). Biological monitoring for Ochratoxin A and Citrinin and their metabolites in urine samples of infants and children in Bangladesh. Mycotoxin Res..

[81] Ali N., Hossain K., Degen G.H. (2018). Blood plasma biomarkers of Citrinin and Ochratoxin A exposure in young adults in Bangladesh. Mycotoxin Res..

[82] Degen G., Ali N., Blaszkewicz M., Hossain K. (2014). First biomonitoring data for the Nephrotoxic Mycotoxins Citrinin and Ochratoxin A in Bangladesh. Toxicol. Lett..

[83] Ali N., Manirujjaman M., Rana S., Degen G.H. (2020). Determination of Aflatoxin M(1)and Deoxynivalenol biomarkers in infants and children urines from Bangladesh. Arch. Toxicol..

[84] Berthiller F., Krska R., Domig K.J., Kneifel W., Juge N., Schuhmacher R., Adam G. (2011). Hydrolytic fate of Deoxynivalenol-3-Glucoside during digestion. Toxicol. Lett..

[85] Nathanail A.V., Syvähuoko J., Malachová A., Jestoi M., Varga E., Michlmayr H., Adam G., Sieviläinen E., Berthiller F., Peltonen K. (2015). Simultaneous determination of major type A and B Trichothecenes, Zearalenone and Certain modified metabolites in Finnish cereal grains with a novel liquid chromatography-tandem mass spectrometric method. Anal. Bioanal. Chem..

[86] Stockmann-Juvala H., Savolainen K. (2008). A review of the toxic effects and mechanisms of action of Fumonisin B1. Hum. Exp. Toxicol.

[87] Van der Westhuizen L., Shephard G.S., Burger H.M., Rheeder J.P., Gelderblom W.C.A., Wild C.P., Gong Y.Y. (2011). Fumonisin B1 as a urinary biomarker of exposure in a maize intervention study among South African subsistence farmers. Cancer Epidemiol. Biomark. Prev..

[88] Van der Westhuizen L., Shephard G.S., Gelderblom W.C.A., Torres O., Riley R.T. (2013). Fumonisin biomarkers in maize eaters and implications for human disease. World Mycotoxin J..

[89] Gormley I.C., Bai Y., Brennan L. (2020). Combining biomarker and self-reported dietary intake data: A review of the state of the art and an exposition of concepts. Stat. Methods Med. Res..

[90] Escrivá L., Font G., Manyes L., Berrada H. (2017). Studies on the presence of mycotoxins in biological samples: An overview. Toxins.

[91] Koller G., Wichmann G., Rolle-Kampczyk U., Popp P., Herbarth O. (2016). Comparison of ELISA and capillary electrophoresis with laser-induced fluorescence detection in the analysis of Ochratoxin A in low volumes of human blood serum. J. Chromatogr. B.

[92] Sangare-Tigori B., Moukha S., Kouadio J.H., Dano D.S., Betbeder A.-M., Achour A., Creppy E.E. (2006). Ochratoxin A in human blood in Abidjan, Cote d’Ivoire. Toxicon.

[93] Muñoz K., Vega M., Rios G., Muñoz S., Madariaga R. (2006). Preliminary study of Ochratoxin A in human plasma in agricultural zones of Chile and its relation to food consumption. Food Chem. Toxicol..

[94] Degen G.H., Mayer S., Blaszkewicz M. (2007). Biomonitoring of ochratoxin A in grain workers. Mycotoxin Res..

[95] Medina Á., Mateo E.M., Roig R.J., Blanquer A., Jiménez M. (2010). Ochratoxin A levels in the plasma of healthy blood donors from Valencia and estimation of exposure degree: Comparison with previous national Spanish data. Food Addit. Contam. Part. A.

[96] Hmaissia Khlifaa K., Ghalib R., Mazigha C., Aounia Z., Machgoula S., Hedhili A. (2012). Ochratoxin A levels in human serum and foods from nephropathy patients in Tunisia: Where are you now?. Exp. Toxicol. Pathol..

[97] Aslam M., Rivzi S.A.H., Beg A.E., Blaszkewicz M., Golka K., Degen G.H. (2012). Analysis of Ochratoxin a blood levels in bladder cancer cases and healthy persons from Pakistan. J. Toxicol. Environ. Health Part. A.

[98] Dohnal V., Dvorák V., Malír F., Ostry V., Roubal T. (2013). A comparison of ELISA and HPLC methods for determination of ochratoxin A in human blood serum in the Czech Republic. Food Chem. Toxicol..

[99] Malir F., Ostry V., Dofkova M., Roubal T., Dvorak V., Dohnal V. (2013). Ochratoxin A levels in blood serum of Czech women in the first trimester of pregnancy and its correspondence with dietary intake of the mycotoxin contaminant. Biomarkers.

[100] Pena A., Seifrtova M. (2006). Estimation of ochratoxin A in portuguese population: New data on the occurrence in human urine by high performance liquid chromatography with fluorescence detection. Food Chem. Toxicol..

[101] Manique R., Pena A., Lino C.M., Moltó J.C., Mañes J. (2008). Ochratoxin A in the morning and afternoon portions of urine from Coimbra and Valencian populations. Toxicon.

[102] Vatinno R., Vuckovic D., Zambonin C.G., Pawliszyn J. (2008). Automated high-throughput method using solid-phase microextraction-liquid chromatography-tandem mass spectrometry for the determination of ochratoxin A in human urine. J. Chromatogr. A.

[103] Ali N., Muñoz K., Degen G.H. (2017). Ochratoxin A and its metabolites in urines of German adults—An assessment of variables in biomarker analysis. Toxicol. Lett..

[104] Egner P.A., Groopman J.D., Wang J.-S., Kensler T.W., Friesen M.D. (2006). Quantification of Aflatoxin-B1-N7-Guanine in human urine by high-performance liquid chromatography and isotope dilution tandem mass spectrometry. Chem. Res. Toxicol..

[105] Warth B., Sulyok M., Berthiller F., Schuhmacher R., Fruhmann P., Hametner C., Adam G., Fröhlich J., Krska R. (2011). Direct quantification of deoxynivalenol glucuronide in human urine as biomarker of exposure to the *Fusarium* mycotoxin deoxynivalenol. Anal. Bioanal. Chem..

[106] Fushimi Y., Takagi M., Uno S., Kokushi E., Nakamura M., Hasunuma H., Shinya U., Deguchi E., Fink-Gremmels J. (2014). Measurement of Sterigmatocystin Concentrations in Urine for Monitoring the Contamination of Cattle Feed. Toxins.

[107] Turner P.C., Ji B.T., Shu X.O., Zheng W., Chow W.-H., Gao Y.T., Hardie L.J. (2011). A biomarker survey of urinary deoxynivalenol in China: The Shanghai women’s health study. Food Addit. Contam. Part A Chem. Anal. Control. Expo. Risk Assess..

[108] Warth B., Sulyok M., Fruhmann P., Berthiller F., Schuhmacher R., Hametner C., Adam G., Fröhlich J., Krska R. (2012). Assessment of human deoxynivalenol exposure using an LC-MS/MS based biomarker method. Toxicol. Lett..

[109] Lattanzio V.M.T., Solfrizzo M., De Girolamo A., Chulze S.N., Torres A.M., Visconti A. (2011). LC-MS/MS characterization of the urinary excretion profile of the mycotoxin deoxynivalenol in human and rat. J. Chromatogr. B.

[110] Silva L.J.G., Pena A., Lino C.M., Fernández M.F., Mañes J. (2010). Fumonisins determination in urine by LC-MS-MS. Anal. Bioanal. Chem..

[111] Andrés F., Zougagh M., Casta G., Ríos A. (2008). Determination of zearalenone and its metabolites in urine samples by liquid chromatography with electrochemical detection using a carbon nanotube-modified electrode. J. Chromatogr. A.

[112] Ali N., Blaszkewicz M., Degen G.H. (2015). Occurrence of the mycotoxin citrinin and its metabolite dihydrocitrinone in urines of German adults. Arch. Toxicol..

[113] Ali N., Blaszkewicz M., Mohanto N.C., Rahman M., Alim A., Hossain K., Degen G.H. (2015). First results on citrinin biomarkers in urines from rural and urban cohorts in Bangladesh. Mycotoxin Res..

[114] Ali N., Blaszkewicz M., Al Nahid A., Rahman M., Degen G.H. (2015). Deoxynivalenol Exposure Assessment for Pregnant Women in Bangladesh. Toxins.

[115] Ali N., Blaszkewicz M., Degen G.H. (2016). Assessment of deoxynivalenol exposure among Bangladeshi and German adults by a biomarker-based approach. Toxicol. Lett..

[116] Muñoz K., Cramer B., Dopstadt J., Humpf H.-U., Degen G.H. (2017). Evidence of Ochratoxin A conjugates in urine samples from infants and adults. Mycotoxin Res..

[117] Ahn J., Kim D., Kim H., Jahng K.-Y. (2010). Quantitative determination of mycotoxins in urine by LC-MS/MS. Food Addit. Contam. Part A.

[118] Rubert J., Soriano J.M., Mañes J., Soler C. (2011). Rapid Mycotoxin analysis in human urine: A pilot study. Food Chem. Toxicol..

[119] Gambacorta S., Solfrizzo H., Visconti A., Powers S., Cossalter A.M., Pinton P., Oswald I.P. (2013). Validation study on urinary biomarkers of exposure for aflatoxin B1, ochratoxin A, fumonisin B1, deoxynivalenol and zearalenone in piglets. World Mycotoxin J..

[120] Song S., Ediage E.N., Wu A., De Saeger S. (2013). Development and application of salting-out assisted liquid/liquid extraction for multi-mycotoxin biomarkers analysis in pig urine with high performance liquid chromatography/tandemmass spectrometry. J. Chromatogr. A.

[121] Ediage E.N., Di Mavungua J.D., Song S., Wu A., Van Peteghem C., De Saeger S. (2012). A direct assessment of mycotoxin biomarkers in human urine samples by liquid chromatography tandem mass spectrometry. Anal. Chim. Acta.

[122] Warth B., Sulyok M., Fruhmann P., Mikula H., Berthiller F., Schuhmacher R., Hametner C., Abia W.A., Adam G., Fröhlich J. (2012). Development and validation of a rapid multi-biomarker liquid chromatography/tandem mass spectrometry method to assess human exposure to mycotoxins: LC/MS/MS method to assess human exposure to mycotoxins. Rapid Commun. Mass Spectrom..

[123] Warth B., Sulyok M., Krska R. (2013). LC-MS/MS-based multibiomarker approaches for the assessment of human exposure to mycotoxins. Anal. Bioanal. Chem..

[124] Solfrizzo M., Gambacorta L., Visconti A. (2014). Assessment of multi-mycotoxin exposure in southern Italy by urinary multi-biomarker determination. Toxins.

[125] Ezekiel C.N., Warth B., Ogara I.M., Abia W.A., Ezekiel V.C., Atehnkeng J., Sulyok M., Turner P.C., Tayo G.O., Krska R. (2014). Mycotoxin exposure in rural residents in northern Nigeria: A pilot study using multi-urinary biomarkers. Environ. Int..

[126] Heyndrickx E., Sioen I., Huybrechts B., Callebaut A., De Henauw S., De Saeger S. (2015). Human biomonitoring of multiple mycotoxins in the belgian population: Results of the BIOMYCO Study. Environ. Int..

[127] Heyndrickx E., Sioen I., Bellemans M., De Maeyer M., Callebaut A., De Henauw S., De Saeger S. (2014). Assessment of mycotoxin exposure in the Belgian population using biomarkers: Aim, design and methods of the BIOMYCO study. Food Addit. Contam. Part A.

[128] Huybrechts B., Martins J.C., Debongnie P., Uhlig S., Callebaut A. (2015). Fast and sensitive LC-MS/MS method measuring human mycotoxin exposure using biomarkers in urine. Arch. Toxicol..

[129] Gerding J., Cramer B., Humpf H. (2014). Determination of mycotoxin exposure in Germany using an LC-MS/MS multibiomarker approach. Mol. Nutr. Food Res..

[130] Gerding J., Ali N., Schwartzbord J., Cramer B., Brown D.L., Degen G.H., Humpf H. (2015). A comparative study of the human urinary mycotoxin excretion patterns in Bangladesh, Germany, and Haiti using a rapid and sensitive LC-MS/MS approach. Mycotoxin Res..

[131] Rodríguez-Carrasco Y., Mañes J., Berrada H., Font G. (2015). Preliminary estimation of Deoxynivalenol excretion through a 24 h pilot study. Toxins.

[132] Rodríguez-Carrasco Y., Moltó J.C., Mañes J., Berrada H. (2014). Development of a GC–MS/MS strategy to determine 15 mycotoxins and metabolites in human urine. Talanta.

[133] Rodríguez-Carrasco Y., Moltó J.C., Mañes J., Berrada H. (2014). Exposure assessment approach through mycotoxin/creatinine ratio evaluation in urine by GC-MS/MS. Food Chem. Toxicol..

[134] Rodríguez-Carrasco Y., Moltó J.C., Mañes J., Berrada H. (2017). Development of microextraction techniques in combination with GC-MS/MS for the determination of mycotoxins and metabolites in human urine. J. Sep. Sci..

[135] Gürbay A., Girgin G., Sabuncuog S.A., Sahin G., Yurdakök M., Yig S., Tekinalp G. (2009). Ochratoxin A: Is it present in breast milk samples obtained from mothers from Ankara, Turkey?. J. Appl. Toxicol..

[136] Afshar P., Shokrzadeh M., Kalhori S., Babaee Z., Saravi S.S.S. (2013). Occurrence of Ochratoxin A and Aflatoxin M1 in human breast milk in Sari, Iran. Food Control.

[137] Massart F., Micillo F., Rivezzi G., Perrone L., Baggiani A., Miccoli M., Meucci V. (2016). Zearalenone screening of human breast milk from the Naples area. Toxicol. Environ. Chem..

[138] Rubert J., León N., Sáez C., Martins C.P.B., Godula M., Yusà V., Mañes J., Soriano J.M., Soler C. (2014). Evaluation of mycotoxins and their metabolites in human breast milk using liquid chromatography coupled to high resolution mass spectrometry. Anal. Chim. Acta.

[139] Andrade P.D., Gomes da Silva J.L., Caldas E.D. (2013). Simultaneous analysis of aflatoxins B1, B2, G1, G2, M1 and ochratoxinA in breastmilk by high-performance liquidchromatography/fluorescence after liquid-liquid extraction with lowtemperature purification (LLE-LTP). J. Chromatogr. A.

[140] Muñoz K., Blaszkewicz M., Degen G.H. (2010). Simultaneous analysis of ochratoxin A and its major metabolite ochratoxin alpha in plasma and urine for an advanced biomonitoring of the mycotoxin. J. Chromatogr. B.

[141] Ali N., Blaszkewicz M., Manirujjaman M., Degen G.H. (2016). Biomonitoring of concurrent exposure to ochratoxin A and citrinin in pregnant women in Bangladesh. Mycotoxin Res..

[142] Blaszkewicz M., Muñoz K., Degen G.H. (2013). Methods for analysis of citrinin in human blood and urine. Arch. Toxicol..

[143] Serrano A.B., Capriotti A.L., Cavaliere C., Piovesana S., Samperi R., Ventura S., Laganà A. (2015). Development of a Rapid LC-MS/MS method for the determination of emerging *Fusarium* mycotoxins Enniatins and Beauvericin in human biological fluids. Toxins.

[144] Camel V., Ouethrani M., Coudray C., Philippe C., Rabot S. (2012). Semi-automated solid-phase extraction method for studying the biodegradation of ochratoxin A by human intestinal microbiota. J. Chromatogr. B.

[145] Hooper D.G., Bolton V.E., Guilford F.T., Straus D.C. (2009). Mycotoxin detection in human samples from patients exposed to environmental molds. Int. J. Mol. Sci..

[146] Cao X., Wu S., Yue Y., Wang S., Wang Y., Tao L., Tian H. (2013). A high-throughput method for the simultaneous determination of multiple mycotoxins in human and laboratory animal biological fluids and tissues by PLE and HPLC-MS/MS. J. Chromatogr. B.

[147] Campbell T.C., Caedo J.P., Bulatao-Jayme J., Salamat L., Engel R.W. (1970). Aflatoxin M1 in human urine. Nature.

[148] Zarba A., Wild C.P., Hall A.J., Montesano R., Hudson G.J., Groopman J.D. (1992). Aflatoxin M1 in human breast milk from The Gambia, West Africa, quantified by combined monoclonal antibody immunoaffinity chromatography and HPLC. Carcinogenesis.

[149] Scholl P.F., Musser S.M., Groopman J.D. (1997). Synthesis and characterization of Aflatoxin B1 Mercapturic acids and their identification in rat urine. Chem. Res. Toxicol..

[150] Groopman J.D., Wild C.P., Hasler J., Junshi C., Wogan G.N., Kensler T.W. (1993). Molecular epidemiology of Aflatoxin exposures: Validation of Aflatoxin-N7-Guanine levels in urine as a biomarker in experimental rat models and humans. Environ. Health Perspect..

[151] Tang L., Tang M., Xu L., Luo H., Huang T., Yu J., Zhang L., Gao W., Cox S.B., Wang J.-S. (2008). Modulation of Aflatoxin bi-omarkers in human blood and urine by green tea polyphenols intervention. Carcinogenesis.

[152] Tang Y., Tang D., Zhang J., Tang D. (2018). Novel quartz crystal microbalance immunodetection of Aflatoxin B-1 coupling cargo-encapsulated Liposome with indicator-triggered displacement assay. Anal. Chim. Acta.

[153] Schwartzbord J.R., Leroy J.L., Severe L., Brown D.L. (2016). Urinary Aflatoxin M1 in Port-Au-Prince and a rural community in north-east Haiti. Food Addit. Contam. Part A Chem Anal. Control. Expo. Risk Assess..

[154] Xue K.S., Cai W., Tang L., Wang J.-S. (2016). Aflatoxin B1-Lysine adduct in dried blood spot samples of animals and humans. Food Chem. Toxicol..

[155] Malir F., Ostry V., Pfohl-Leszkowicz A., Malir J., Toman J. (2016). Ochratoxin A: 50 years of research. Toxins.

[156] Malir F., Louda M., Ostry V., Toman J., Ali N., Grosse Y., Malirova E., Pacovsky J., Pickova D., Brodak M. (2019). Anal-yses of biomarkers of exposure to Nephrotoxic Mycotoxins in a cohort of patients with renal tumours. Mycotoxin Res..

[157] Studer-Rohr I., Schlatter J., Dietrich D.R. (2000). Kinetic parameters and intraindividual fluctuations of Ochratoxin A plasma levels in humans. Arch. Toxicol..

[158] Scott P.M. (2005). Biomarkers of human exposure to Ochratoxin, A. Food Addit. Contam. Suppl..

[159] Degen G.H. (2016). Are we ready to estimate daily Ochratoxin A intake based on urinary concentrations?. Environ. Int..

[160] Coronel M.B., Marin S., Tarragó M., Cano-Sancho G., Ramos A.J., Sanchis V. (2011). Ochratoxin A and its metabolite Ochratoxin Alpha in urine and assessment of the exposure of inhabitants of Lleida, Spain. Food Chem. Toxicol..

[161] Han Z., Tangni E.K., Di Mavungu J.D., Vanhaecke L., De Saeger S., Wu A., Callebaut A. (2013). In vitro Glucuronidation of Ochratoxin A by rat liver microsomes. Toxins.

[162] Wu Q., Dohnal V., Huang L., Kuca K., Wang X., Chen G., Yuan Z. (2011). Metabolic pathways of Ochratoxin, A. CDM.

[163] Hult K., Pleština R., Habazin-Novak V., Radić B., Čeović S. (1982). Ochratoxin A in human blood and Balkan endemic nephropathy. Arch. Toxicol..

[164] Hult K., Fuchs R., Peraica M., Pleština R., Čeović S. (1984). Screening for Ochratoxin A in blood by flow injection analysis. J. Appl. Toxicol..

[165] Maaroufi K., Achour A., Hammami M., El May M., Betbeder A., Ellouz F., Creppy E., Bacha H. (1995). Ochratoxin A in human blood in relation to Nephropathy in Tunisia. Hum. Exp. Toxicol..

[166] Beker D., Radić B. (1991). Fast determination of Ochratoxin A in serum by liquid chromatography: Comparison with enzymic spectrofluorimetric method. J. Chromatogr. B Biomed. Sci. Appl..

[167] Cramer B., Osteresch B., Munoz K.A., Hillmann H., Sibrowski W., Humpf H.-U. (2015). Biomonitoring using dried blood spots: Detection of ochratoxin A and its degradation product 2′R-ochratoxin A in blood from coffee drinkers. Mol. Nutr. Food Res..

[168] Osteresch B., Cramer B., Humpf H.U. (2016). Analysis of ochratoxin A in dried blood spots—Correlation between venous and finger-prick blood, the influence of hematocrit and spotted volume. J. Chromatogr. B Anal. Technol. Biomed. Life Sci..

[169] Pestka J.J., Steinert B.W., Chu F.S. (1981). Enzyme-linked immunosorbent assay for detection of Ochratoxin, A. Appl. Environ. Microbiol..

[170] Ueno Y., Maki S., Lin J., Furuya M., Sugiura Y., Kawamura O. (1998). A 4-year study of plasma Ochratoxin A in a selected population in Tokyo by immunoassay and immunoaffinity column-linked HPLC. Food Chem. Toxicol..

[171] Mycotoxin Mixtures in Food and Feed: Holistic, Innovative, Flexible Risk Assessment Modelling Approach: MYCHIF. https://efsa.onlinelibrary.wiley.com/doi/pdf/10.2903/sp.efsa.2020.EN-1757.

[172] Orti D.L., Hill R.H., Liddle J.A., Needham L.L., Vickers L. (1986). High performance liquid chromatography of Mycotoxin Me-tabolites in human urine. J. Anal. Toxicol..

[173] Cao J., Kong W., Zhou S., Yin L., Wan L., Yang M. (2013). Molecularly imprinted polymer-based solid phase clean-up for analysis of Ochratoxin A in beer, red wine, and grape juice: Sample preparation. J. Sep. Sci..

[174] Yu J.C.C., Lai E.P.C. (2010). Molecularly imprinted polymers for Ochratoxin A extraction and analysis. Toxins.

[175] De Ruyck K., Huybrechts I., Yang S., Arcella D., Claeys L., Abbeddou S., De Keyzer W., De Vries J., Ocke M., Ruprich J. (2020). Mycotoxin exposure assessments in a multi-center European validation study by 24-hour dietary recall and bio-logical fluid sampling. Environ. Int..

[176] Metzler M., Pfeiffer E., Hildebrand A. (2010). Zearalenone and its metabolites as endocrine disrupting chemicals. World Mycotoxin J..

[177] Miles C.O., Erasmuson A.F., Wilkins A.L., Towers N.R., Smith B.L., Garthwaite I., Scahill B.G., Hansen R.P. (1996). Ovine metabolism of zearalenone to α-Zearalanol (Zeranol). J. Agric. Food Chem..

[178] Pfeiffer E., Hildebrand A., Damm G., Rapp A., Cramer B., Humpf H.-U., Metzler M. (2009). Aromatic hydroxylation is a major metabolic pathway of the Mycotoxin Zearalenone in vitro. Mol. Nutr. Food Res..

[179] Bravin F., Duca R., Balaguer P., Delaforge M. (2009). In vitro Cytochrome P450 formation of a Mono-Hydroxylated metabolite of Zearalenone Exhibiting estrogenic activities: Possible occurrence of this metabolite in vivo. IJMS.

[180] Abia W.A., Warth B., Sulyok M., Krska R., Tchana A., Njobeh P.B., Turner P.C., Kouanfack C., Eyongetah M., Dutton M. (2013). Bio-monitoring of Mycotoxin exposure in Cameroon using a urinary multi-biomarker approach. Food Chem. Toxicol..

[181] Pfeiffer E., Hildebrand A., Mikula H., Metzler M. (2010). Glucuronidation of Zearalenone, Zeranol and four metabolites in vitro: Formation of Glucuronides by various microsomes and human UDP-Glucuronosyltransferase Isoforms. Mol. Nutr. Food Res..

[182] Warth B., Petchkongkaew A., Sulyok M., Krska R. (2014). Utilising an LC-MS/MS-based multi-biomarker approach to assess mycotoxin exposure in the Bangkok metropolitan area and surrounding provinces. Food Addit. Contam. Part A Chem. Anal. Control Expo. Risk Assess..

[183] Warth B., Preindl K., Manser P., Wick P., Marko D., Buerki-Thurnherr T. (2019). Transfer and metabolism of the Xenoestrogen Zearalenone in human perfused placenta. Environ. Health Perspect..

[184] Udovicki B., Audenaert K., De Saeger S., Rajkovic A. (2018). Overview on the mycotoxins incidence in Serbia in the period 2004–2016. Toxins.

[185] Pestka J.J. (2010). Deoxynivalenol: Mechanisms of action, human exposure, and toxicological relevance. Arch. Toxicol..

[186] Pestka J.J. (2010). Deoxynivalenol-induced proinflammatory gene expression: Mechanisms and pathological sequelae. Toxins.

[187] Pestka J.J., Smolinski A.T. (2005). Deoxynivalenol: Toxicology and potential effects on humans. J. Toxicol. Environ. Health B.

[188] Meky F.A., Turner P.C., Ashcroft A.E., Miller J.D., Qiao Y.-L., Roth M.J., Wild C.P. (2003). Development of a urinary biomarker of human exposure to Deoxynivalenol. Food Chem. Toxicol..

[189] Uhlig S., Ivanova L., Fæste C.K. (2013). Enzyme-assisted synthesis and structural characterization of the 3-, 8-, and 15-Glucuronides of Deoxynivalenol. J. Agric. Food Chem..

[190] Uhlig S., Ivanova L., Fæste C.K. (2016). Correction to enzyme-assisted synthesis and structural characterization of the 3-, 8-, and 15-Glucuronides of Deoxynivalenol. J. Agric. Food Chem..

[191] Warth B., Del Favero G., Wiesenberger G., Puntscher H., Woelflingseder L., Fruhmann P., Sarkanj B., Krska R., Schuhmacher R., Adam G. (2016). Identification of a novel human Deoxynivalenol metabolite enhancing proliferation of intestinal and urinary bladder cells. Sci. Rep..

[192] Nagl V., Schatzmayr G. (2015). Deoxynivalenol and its masked forms in food and feed. Curr. Opin. Food Sci..

[193] Nagl V., Schwartz H., Krska R., Moll W.-D., Knasmüller S., Ritzmann M., Adam G., Berthiller F. (2012). Metabolism of the masked mycotoxin Deoxynivalenol-3-Glucoside in rats. Toxicol. Lett..

[194] Fruhmann P., Warth B., Hametner C., Berthiller F., Horkel E., Adam G., Sulyok M., Krska R., Fröhlich J. (2012). Synthesis of Deoxynivalenol-3-ß-D-O-Glucuronide for its use as biomarker for dietary Deoxynivalenol exposure. World Mycotoxin J..

[195] EFSA Panel on Contaminants in the Food Chain (CONTAM) (2011). Scientific opinion on the risks for animal and public health related to the presence of T-2 and HT-2 toxin in food and feed. EFSA J..

[196] Wu Q., Dohnal V., Huang L., Kuča K., Yuan Z. (2010). Metabolic pathways of trichothecenes. Drug Metab. Rev..

[197] Trombete F., Barros A., Vieira M., Saldanha T., Venancio A., Fraga M. (2016). Simultaneous determination of deoxynivalenol, deoxynivalenol-3-glucoside and nivalenol in wheat grains by HPLC-PDA with immunoaffinity column cleanup. Food Anal. Methods.

[198] Hsia C.C., Wu Z.Y., Li Y.S., Zhang F., Sun Z.T. (2004). Nivalenol, a main *Fusarium* toxin in dietary foods from high-risk areas of cancer of esophagus and gastric cardia in China, induced benign and malignant tumors in mice. Oncol. Rep..

[199] Gonzalez-Arias C.A., Marin S., Sanchis V., Ramos A.J. (2013). Mycotoxin bioaccessibility/absorption assessment using in vitro digestion models: A review. World Mycotox J..

[200] The EFSA Panel on Contaminants in the Food Chain (CONTAM) (2017). Scientific Opinion on the appropriateness to set a group health based guidance value for nivalenol and its modified forms. EFSA J..

[201] Tolosa J., Graziani G., Gaspari A., Chianese D., Ferrer E., Ma˜nes J., Ritieni A. (2017). Multi-mycotoxin analysis in durum wheat pasta by liquid chromatography coupled to quadrupole orbitrap mass spectrometry. Toxins.

[202] Alassane-Kpembi I., Gerez J.R., Cossalter A.M., Neves M., Laffitte J., Naylies C., Oswald I.P. (2017). Intestinal toxicity of the type B trichothecene mycotoxin fusarenon-X: Whole transcriptome profiling reveals new signaling pathways. Sci. Rep..

[203] IARC (1993). Mycotoxins, IARC Monographs on the Evaluation of Carcinogenic Risk of Chemicals to Humans.

[204] Saengtienchai T., Poapolathep S., Isariyodom S., Ikenaka Y., Ishizuka M., Poapolathep A. (2014). Toxicokinetics and tissue depletion of Fusarenon-X and its metabolite nivalenol in piglets. Food Chem. Toxicol..

[205] Poapolathep A., Poapolathep S., Sugita-Konishi Y., Imsilp K., Tassanawat T., Sinthusing C., Kumagai S. (2008). Fate of fusarenon-X in broilers and ducks. Poult. Sci..

[206] Poapolathep A., Sugita-Konishi Y., Doi K., Kumagai S. (2003). The fates of trichothecene mycotoxins, nivalenol and fusarenon-X, in mice. Toxicon.

[207] Vidal A., Mengelers M., Yang S., De Saeger S., De Boevre M. (2018). Mycotoxin biomarkers of exposure: A comprehensive review. Compr. Rev. Food Sci. Food Saf..

[208] Chen C., Riley R.T., Wu F. (2018). Dietary fumonisin and growth impairment in children and animals: A review. Compr. Rev. Food Sci. Food Saf..

[209] Gong Y.Y., Torres-Sanchez L., Lopez-Carrillo L., Peng J.H., Sutcliffe A.E., White K.L., Humpf H.-U., Turner P.C., Wild C.P. (2008). Association between tortilla consumption and human urinary fumonisin B1 Levels in a Mexican population. Cancer Epidemiol. Biomark. Prev..

[210] Riley R.T., Torres O., Showker J.L., Zitomer N.C., Matute J., Voss K.A., Gelineau-van Waes J., Maddox J.R., Gregory S.G., Ashley-Koch A.E. (2012). The kinetics of urinary Fumonisin B_1_ excretion in humans consuming maize-based diets. Mol. Nutr. Food Res..

[211] Wangia R.N., Githanga D.P., Xue K.S., Tang L., Anzala O.A., Wang J.-S. (2019). Validation of urinary sphingolipid metabolites as biomarker of effect for fumonisins exposure in Kenyan children. Biomarkers.

[212] Ediage E.N., Di Mavungu J.D., Song S., Sioen I., De Saeger S. (2013). Multimycotoxin analysis in urines to assess infant exposure: A Case study in Cameroon. Environ. Int..

[213] Sulyok M., Krska R., Schuhmacher R. (2010). Application of an LC–MS/MS based multi-mycotoxin method for the semi-quantitative determination of mycotoxins occurring in different types of food infected by moulds. Food Chem..

[214] Sulyok M., Berthiller F., Krska R., Schuhmacher R. (2006). Development and validation of a liquid chromatography/tandem mass spectrometric method for the determination of 39 mycotoxins in wheat and maize. Rapid Commun. Mass Spectrom..

[215] Gurusankar R., Yenugadhati N., Krishnan K., Hays S., Haines D., Zidek A., Kuchta S., Kinniburgh D., Gabos S., Mattison D. (2017). The role of human biological monitoring in health risk assessment. Int. J. Risk Assess. Manag..

[216] Shephard G.S. (2009). Aflatoxin analysis at the beginning of the twenty-first century. Anal. Bioanal. Chem..

[217] Al-Jaal B.A., Jaganjac M., Barcaru A., Horvatovich P., Lati A. (2019). Aflatoxin, fumonisin, ochratoxin, zearalenone and deoxynivalenol biomarkers in human biological fluids: A systematic literature review, 2001–2018. Food Chem. Toxicol..

[218] Cuadros-Rodríguez L., Bagur-González M.G., Sánchez-Viñas M., González Casado A., Gómez-Sáez A.M. (2007). Principles of analytical calibration/quantification for the separation sciences. J. Chromatogr. A.

[219] Aggett P.J., Antoine J.-M., Asp N.-G., Bellisle F., Contor L., Cummings J.H. (2005). PASSCLAIM: Process for the assessment of scientific support for claims on foods. Eur. J. Nutr..

[220] Dragsted L.O., Gao Q., Scalbert A., Vergères G., Kolehmainen M., Manach C., Brennan L., Afman L.A., Wishart D.S., Andres Lacueva C. (2018). Validation of biomarkers of food intake—Critical assessment of candidate biomarkers. Genes Nutr..

[221] Chen L., Yu M., Wu Q., Peng Z., Wang D., Kuca K., Yao P., Yan H., Nussler A.K., Liu L. (2017). Gender and geographical variability in the exposure pattern and metabolism of deoxynivalenol in humans: A review. J. Appl. Toxicol..

[222] Mitropoulou A., Gambacorta L., Warensjö Lemming E., Solfrizzo M., Olsen M. (2018). Extended evaluation of urinary multi-biomarker analyses of mycotoxins in Swedish adults and children. World Mycotoxin J..

[223] Lemming E.W., Montes A.M., Schmidt J., Cramer B., Humpf H.U., Moraeus L., Olsen M. (2020). Mycotoxins in blood and urine of Swedish adolescents—Possible associations to food intake and other background characteristics. Mycotoxin Res..

[224] Boon P.E., Bakker R., van Klaveren J.D., van Rossum C.T.M. Risk Assessment of the Dietary Exposure to Contaminants and Pesticide Residues in Young Children in the Netherlands, 2009 (RIVM rapport; No. 350070002). https://edepot.wur.nl/17759.

[225] Sprong R.C., de Wit-Bos L., Zeilmaker M.J., Alewijn M., Castenmiller J.J.M., Mengelers M.J.B. (2016). A mycotoxin-dedicated total diet study in the Netherlands in 2013: Part I—Design. World Mycotoxin J..

[226] European Food Safety Authority (2011). Use of the EFSA comprehensive European food consumption database in exposure assessment. EFS2.

[227] De Nijs M., Pereboom-de Fauw D.P.K.H., van Dam R.C.J., de Rijk T.C., van Egmond H.P., Mol H.J.G.J. (2013). Development and validation of an LC-MS/MS method for the detection of Phomopsin A in lupin and lupin-containing retail food samples from the Netherlands. Food Addit. Contam. Part A Chem. Anal. Control. Expo. Risk Assess..

[228] López P., Venema D., de Rijk T., de Kok A., Scholten J.M., Mol H.G.J., de Nijs M. (2016). Occurrence of alternaria toxins in food products in The Netherlands. Food Control.

[229] Sanders M., Landschoot S., Audenaert K., Haesaert G., Eeckhout M., De Saeger S. (2014). Deoxynivalenol content in wheat dust versus wheat grain: A comparative study. World Mycotoxin J..

[230] Müller M.E.H., Korn U. (2013). Alternaria Mycotoxins in wheat—A 10 years survey in the northeast of Germany. Food Control.

[231] European Food Safety Authority (2013). Standard sample description ver. 2.0. EFS2.

[232] De Rijk T.C., van Egmond H.P., van der Fels-Klerx H.J., Herbes R., de Nijs M., Samson R.A., Slate A.B., van der Spiegel M. (2015). A Study of the 2013 Western European issue of Aflatoxin contamination of maize from the Balkan area. World Mycotoxin J..

[233] WHO Towards a Harmonised Total Diet Study Approach: A Guidance Document. http://www.who.int/foodsafety/publications/tds_guidance/en/.

[234] European Food Safety Authority (EFSA), Food and Agriculture Organization of the United Nations (FAO), World Health Organization (WHO) (2011). State of the art on total diet studies based on the replies to the EFSA/FAO/WHO questionnaire on national total diet study approaches. EFS3.

[235] Jekel A.A., van Egmond H.P. (2014). Determination of T-2/HT-2 toxins in duplicate diets in The Netherlands by GC-MS/MS: Method development and estimation of human exposure. World Mycotoxin J..

[236] FAO (2018). Dietary Assessment: A Resource Guide to Method Selection and Application in Low Resource Settings.

[237] Cressey P.J., Reeve J. (2013). Dietary exposure and risk assessment for aflatoxins in New Zealand. World Mycotoxin J..

[238] Boon P.E., Probabilistic Dietary Exposure Models RIVM Letter Report 2015-0191. https://www.rivm.nl/bibliotheek/rapporten/2015-0191.pdf.

